# Retraction: Estimation of progression of multi-state chronic disease using the Markov model and prevalence pool concept

**DOI:** 10.1186/1472-6947-9-45

**Published:** 2009-10-20

**Authors:** Hui-Chuan Shih, Pesus Chou, Chi-Ming Liu, Tao-Hsin Tung

**Affiliations:** 1 Department of Nursing, Kaohsiung Armed Forces General Hospital, Kaohsiung, Taiwan, Republic of China; 2 Community Medicine Research Center and Institute of Public Health, National Yang-Ming University, Taipei, Taiwan, Republic of China; 3 Department of Medical Research and Education, Cheng Hsin Rehabilitation Medical Center, Taipei, Taiwan, Republic of China; 4 Faculty of Public Health, School of Medicine, Fu-Jen Catholic University, Taipei, Taiwan, Republic of China

## Abstract

This article [1] has been retracted because the Editors are unable to ensure the scientific veracity of the findings or the ethical conduct of the authors despite an extensive investigation.

## 

This article [1] has been retracted because the Editors are unable to ensure the scientific veracity of the findings or the ethical conduct of the authors despite an extensive investigation.

## Pre-publication history

The pre-publication history for this paper can be accessed here:

http://www.biomedcentral.com/1472-6947/9/45/prepub
